# Analysis on the application of FMEA in ‘instrument and equipment surface cleaning and disinfection’ in hospitals based on standardization and cleaning and disinfection information system management

**DOI:** 10.3389/fpubh.2024.1444721

**Published:** 2024-09-25

**Authors:** Jing Zheng, Ling Wang, Yihai Fang, Xuejun Xu, Li Hu

**Affiliations:** ^1^Infection Management Department, The Pearl River Hospital, Southern Medical University, Guangzhou, Guangdong, China; ^2^Equipment Department, Pearl River Hospital, Southern Medical University, Guangzhou, Guangdong, China; ^3^Medical Insurance Affairs Department, Pearl River Hospital, Southern Medical University, Guangzhou, Guangdong, China; ^4^Outpatient Department of Stomatology, Pearl River Hospital, Southern Medical University, Guangzhou, Guangdong, China

**Keywords:** standardization, cleaning and disinfection information system, instruments and equipment, application, FMEA preface

## Abstract

**Purpose:**

To analyze the application of ‘instrument and equipment surface cleaning and disinfection’ in hospitals based on standardization and the management of cleaning and disinfection information systems.

**Methods:**

Employees and all cleaning and disinfected instruments and equipment from 56 inpatient departments in our hospital were selected as the subjects of observation. The period before the intervention (January 2023) was designated as the control group, while the period after the intervention (July 2023) was designated as the study group. In the control group, the instruments and equipment under routine management were disinfected. The research team applied the Failure Mode and Effects Analysis (FMEA) method to clean and disinfect the surfaces of instruments and equipment on the basis of standardization and cleaning and disinfection information system management. Employees’ theoretical knowledge points and operational skill scores before and after the intervention were compared and evaluated. The changes in the risk priority coefficient (RPN) values of high-risk factors were analyzed. Fifty-six clinical medical staff from 56 inpatient departments in the hospital were selected to evaluate the clinical satisfaction of the cleaning and disinfection management of instruments and equipment before and after the intervention, and the clinical satisfaction of the two groups was compared.

**Results:**

The scores of theoretical knowledge and operational skills of the staff in the research group were significantly higher than those in the control group. The passing rates of theoretical knowledge and operational skills in the control group and the research group were 44.64 and 94.64% respectively, and 55.36 and 96.43%, respectively. The qualified rate of theoretical knowledge and operational skills of staff in the study group was significantly higher than that in the control group (*p* < 0.05). The RPN scores of medical personnel, environment, system and system guarantee factors in the control group were 80, 80, 80, and 100, respectively. The RPN scores of medical personnel factors, environmental factors, system factors and system guarantee factors in the research group were 6, 24, 24, and 36, respectively.

**Conclusion:**

Through standardization and cleaning and disinfection information system management, the theoretical knowledge and technical operation capabilities of cleaning can be effectively improved.

## 1 Introduction

With the rapid development of medical technology, the types of medical instruments and equipment continue to increase. Hospitals rely more on the application of medical equipment (1, 2), but ignore the refined management of equipment. Due to the long-term high-load use of instruments and equipment to assist clinical examination and treatment, but lack of standardized layout, cleaning and disinfection, the surface of the instruments and equipment is easily contaminated, becoming an important medium for the survival and spread of hospital infection pathogens (3, 4). As a result, how to effectively clean and disinfect medical equipment has become the focus of current medical and health institutions. In clinical practice, staff typically perform routine surface cleaning and disinfection of medical devices. However, due to insufficient management standards, the failure rate of equipment cleaning and disinfection is very high. The chaotic arrangement of the number of instruments and equipment affects clinical rescue work (5, 6). In response to the increasing emphasis on hospital infections in recent years, local and national standards have been issued to standardize the management requirements for the cleaning and disinfection of medical devices.

To address these challenges, we have integrated Failure Mode and Effects Analysis (FMEA) into our approach. FMEA is a widely recognized risk management method used to analyze potential failure modes within a specific process. By classifying and sorting failure modes, and implementing preventive and corrective measures for high-risk scenarios, FMEA helps in formulating a robust management system that enhances quality control (7, 8). Therefore, based on standardization and combined with informatized cleaning and disinfection information system management, we have formulated more standardized management processes, achieved cross-department collaboration, and improved efficiency (9, 10). This experiment selected 56 employees from 56 inpatient departments of our hospital, and all cleaning and disinfection instruments and equipment within these departments were used as observation objects, aiming to realize the application of “instrument and equipment surface cleaning and disinfection” based on standardization and the management of hospital cleaning and disinfection information systems.

## 2 Materials and methods

### 2.1 General information

We selected employees and all cleaning and disinfection instruments and equipment from 56 inpatient departments in our hospital as the observation objects for this study (Including patient monitoring equipment, infusion pumps and other equipment). Inclusion criteria for instruments and equipment: All instruments are medical instruments that have been used in our hospital for a long time, are intact and can be used normally. The study lasted for a total of 6 months, with the period before the intervention (January 2023) was set as the control group, and the period after the intervention (July 2023) was set as the research group. All aspects of the study involving human participants, were reviewed and approved by the hospital’s ethics committee.

### 2.2 Method

The steps of the methodology are based on the FMEA method. We performed FMEA by following a systematic approach that included the following steps: Identification of Failure Modes, Assessment of Effects, Determination of Causes, Risk Evaluation, Implementation of Preventive Measures, Monitoring and Review.

The control group was given routine management of instrument and equipment disinfection: the surfaces of medical instruments were cleaned and disinfected in accordance with the requirements of the “Management Specifications for Cleaning and Disinfection of Environmental Surfaces in Medical Institutions.” These specifications outline essential procedures including: (1) Pre-Cleaning: Removing visible debris from surfaces before applying disinfectants. (2) Disinfection: Applying appropriate disinfectants that are effective against common pathogens. (3) Contact Time: Ensuring that disinfectants remain in contact with surfaces for the recommended duration to achieve effective microbial reduction. (4) Cleaning Frequency: Regular cleaning and disinfection schedules to maintain hygiene standards. (5) Documentation: Recording cleaning and disinfection activities to ensure compliance and facilitate audits.

The research team cleans and disinfects the surfaces of instruments and equipment on the basis of standardization and cleaning and disinfection information system management: Standardized cleaning and disinfection: refer to the “Technical Specifications for Disinfection of Medical Institutions,” “Hygienic Standards for Hospital Disinfection,” “Hospital Infections in Intensive Care Units” “Code for Situation Management” requires the cleaning and disinfection of medical device surfaces: (1) Continuously used instruments and equipment such as ECG monitors should be cleaned and disinfected at any time when contaminated, and terminal disinfection should be carried out after use; (2) Equipment that is cross-used by patients, such as electrocardiographs, infusion pumps, sphygmomanometers, thermometers, and ultrasound machines, should be cleaned and disinfected immediately after contact with patient areas, rather than once a week when in direct contact with patient areas; (3) Computers used in the medical area should be cleaned and disinfected 1–2 times a day; (4) For sufferers with drug-resistant infections or medical devices continuously used by infected sufferers, the frequency of surface cleaning and disinfection should be more than once a day. If an infection outbreak or suspected outbreak occurs, the frequency of cleaning and disinfection needs to be increased; (5) The storage area or cabinet of medical equipment must be kept clean and dry, and should be cleaned and disinfected once a week. If contaminated, please clean and disinfect at any time.

Cleaning and disinfection information system management: (1) Team formation: Establishing the “Instrument Equipment Surface Cleaning and Disinfection” project team. Team members include eight staff members from the Infection Management, Nursing and Facilities Departments. All members have received unified training in FMEA and are proficient in this knowledge. (2) Risk identification: Based on actual work experience, team members find relevant links and factors that May lead to unqualified cleaning and disinfection through brainstorming and reviewing relevant literature. (3) Risk analysis and evaluation: Based on the relevant links and factors that May lead to unqualified cleaning and disinfection, develop a “Risk Assessment Form” and scoring standards. Team members evaluate the severity of each risk point based on the scoring criteria, (Severity S), risk occurrence ratio (Occurrence, O), and risk detection (Occurrence, D). The values of S, O, and D range from 1 to 5 points. The risk priority number (RPN) value of each project failure mode (RPN = S × O × D) is calculated and sorted according to the score. The top 4 items are prioritized for risk response (see Table 1).

**Table 1:** Four major risk environments and factors in this study.

Link	Factor
Medical staff factors	weak knowledge
Environmental factor	There are a large number of instruments and equipment, arranged in a chaotic manner, and there is no unified standard.
System factors	Manual registration requires a lot of work and is not traceable
System assurance factors	Relevant systems, norms, processes, standards, plans, etc. have not yet been established, revised and improved

Cooperating with the Nursing Department and Equipment Department to continuously improve and adjust the system during the intensive care unit application process to deal with risks: (1) Environmental factors: standardizing the placement area of instruments and equipment in each department of the hospital, standardizing the size, quantity statistics and pasting position of signs, and standardizing cleaning signs and location of cleaning items to minimize the risk of cleaning cross-contamination. (2) Medical personnel factors: Systematic training and assessment of equipment cleaning and disinfection personnel. The director of the Department of Infectious Diseases of our hospital gave a special lecture, focusing on the technical specifications of disinfection. The head nurse explained the relevant knowledge points of “How to Dispose of Reused Medical Equipment” and established an “Instrument Equipment Cleaning and Disinfection Management Group,” which can upload videos of equipment disinfection standards and precautions, and conduct disinfection and cleaning questionnaires and skill assessments for employees. (3) System factors: The Infection Management Department and the Equipment Department are jointly responsible for establishing a design information system that includes tracking the cleaning and disinfection status of each medical device, along with subsequent stages such as maintenance and usage, to reduce clinical workload and improve the registration completion rate. (4) System guarantee factors: The Infection Management Department has formulated the “Instrument and Equipment Surface Cleaning and Disinfection Management System” and operating procedures, which standardizes the surface cleaning and disinfection procedures for equipment across various types and conditions. At the same time, it is stipulated that relevant knowledge training should be conducted once a week, and assessments and system assessments should be conducted monthly to improve employees’ theoretical knowledge and operational skills in the cleaning and disinfection of medical devices.

Establishing a supervision and feedback mechanism: The Department of Infectious Diseases and the Nursing Department jointly conduct random sampling of the cleaned and disinfected instruments and equipment throughout the hospital on a regular basis. At the same time, the results were fed back, the causes were analyzed, continuous quality improvement was carried out, and the implementation rate was improved through random inspection sampling and supervision feedback, ultimately improving the qualification ratio of surface cleaning and disinfection of instruments and equipment.

### 2.3 Observation indicators

Before and after the management intervention, each item was scored out of 100 points. Higher scores indicated greater theoretical knowledge and operational skills of the staff in cleaning and disinfecting instruments and equipment.

Evaluation of changes in RPN values for four high-risk factors was performed by 8 FMEA team members, who scored the three dimensions—Severity (S), Occurrence (O), and Detection (D)—for each risk point. The average RPN values for each failure mode were then calculated and compared between the research group and the control group of employees.

The evaluation of disinfection and cleaning effects involved random inspections and monitoring of medical instruments and equipment both in use and on standby across 56 inpatient departments. Each group conducted random inspections of 112 instruments and equipment. Instruments were considered qualified if the number of bacterial colonies was ≤200 CFU/100 cm^2^. The disinfection and cleaning qualification ratios of the two groups were compared.

For the clinical satisfaction evaluation, 56 clinical medical staff from 56 inpatient departments assessed the cleaning and disinfection management of instruments and equipment. The evaluation covered aspects such as cleaning effectiveness, ease of use, cleaning and disinfection time, and information registration status. The total score was out of 100 points, with ratings categorized as very satisfied (score ≥ 80 points), relatively satisfied (score between 60 and 79 points), and dissatisfied (score < 60 points). The clinical satisfaction of the control group and the research group was compared.

### 2.4 Statistical methods

The experimental data were analyzed using SPSS 20.0 software. Measurement data, such as age, theoretical knowledge scores, and RLU values, were represented as x̄ ± *s* and analyzed using the *t*-test. Categorical data, including gender, education level, and satisfaction, were expressed as percentages and analyzed using the χ^2^ test. Statistical significance was set at *p* < 0.05.

## 3 Results

### 3.1 Comparison of theoretical knowledge and skill operation scores of two groups of employees

The scores of theoretical knowledge and operational skills of the staff in the research group were significantly higher than those in the control group. The passing rates for theoretical knowledge were 44.64% in the control group and 94.64% in the research group. For operational skills, the rates were 55.36% in the control group and 96.43% in the research group. The passing ratios for theoretical knowledge and operational skills in the research group were significantly higher than those in the control group (*p* < 0.05). Please refer to Table 2.

**Table 2:** Comparison of theoretical knowledge and skill operation scores of two groups of staff [*n* (%) (x̄ ± *s*)].

Group	Example	Theoretical knowledge		Operation skills	
		Score	Pass rate	Score	Pass rate
Control group	56	77.79 ± 1 0.33	25 (44.64)	8 1.52 ± 14.04	31 (55.36)
Research team	56	90.30 ± 6.04	53 (94.64)	91.68 ± 5.97	54 (96.43)
*t*		7.828	33.110	4.985	25.816
*P*		<0.001	<0.001	<0.001	<0.001

t: the *t*-value; P, the *p*-value.

### 3.2 Analysis of RPN scores for disinfection and cleaning between two groups

In the control group, the RPN scores of medical personnel factors, environmental factors, system factors, and institutional guarantee factors were 80, 80, 80, and 100, respectively. The RPN scores of medical personnel factors, environmental factors, system factors and institutional guarantee factors of the research group were 6, 24, 24 and 36, respectively. The RPN scores of medical staff factors, environmental factors, system factors, and institutional security factors in the study group were all significantly lower than those in the control group, and the differences were statistically significant (*p* < 0.05). See Table 3.

**Table 3:** Analysis of disinfection and cleaning RPN scores of two groups.

Factor	Group	S	O	D	RPN
Medical staff factors	Control group	4	5	4	80
	Research team	1	2	3	6
Environmental factor	Control group	4	5	4	80
	Research team	2	4	3	24
System factors	Control group	4	5	4	80
	Research team	2	3	4	24
System assurance factors	Control group	4	5	5	100
	Research team	3	3	4	36

### 3.3 Comparison of the cleaning and disinfection qualification rates of the two groups of instruments and equipment

The cleaning and disinfection qualification ratios for the control group and the research group were 55.36 and 87.50%, respectively. The qualified rate of cleaning and disinfection of instruments and equipment in the research group was significantly higher than in the control group (*p* < 0.05). See Table 4.

**Table 4:** Comparison of the cleaning and disinfection qualification rates of the two groups of instruments and equipment [*n* (%)].

Group	*n*	Qualified	Not qualified
Control group	112	62 (55.36)	50 (44.64)
Research team	112	98 (87.50)	14 (12.50)
*X* ^2^		28.350	
*P*		< 0.001	

X^2^, the chi-square statistic; P, the *P*-value.

### 3.4 Clinical staff satisfaction evaluation

The clinical satisfaction rate for the control group and the research group were 76.79 and 91.07%, respectively. The clinical satisfaction in the research group was significantly higher than in the control group, with a statistically significant difference (*p* < 0.05). See Table 5.

**Table 5:** Comparison of Clinical Staff Satisfaction Between the Two Groups [*n* (%)].

Group		Very satisfied	Quite satisfied	Not satisfied	Clinical satisfaction
Control group	56	15 (26.79)	28 (50.00)	13 (23.21)	43 (76.79)
Research team	56	20 (35.71)	31 (55.36)	5 (8.93)	51 (91.07)
*X* ^2^					4.236
*P*					0.040

X^2^, the chi-square statistic; P, the *P*-value.

## 4 Discussion

With the rapid development of medical technology in recent years, the application scope of medical equipment is becoming more and more extensive. However, long-term rotation of medical equipment and insufficient cleaning and disinfection May increase the risk of nosocomial infection, which not only increases the patient’s hospitalization time and treatment difficulty, but also seriously endangers the patient’s life (11, 12). As a result, effective cleaning and disinfection of medical equipment is crucial for reducing hospital infections.

Clinical routine requires hospital staff to clean and disinfect instruments and equipment. However, due to insufficient training, some staff May perform irregular cleaning and disinfection operations and disorderly placement of instruments and equipment, which will have a negative impact on efficient medical operations and hospital disinfection management (13, 14). With the deepening of medical studies in recent years, the focus of scholars’ research on nosocomial infections has gradually shifted to nosocomial infections caused by difficult or incomplete disinfection of medical equipment. Therefore, China has successively issued various disinfection management documents to improve the quality of cleaning and disinfection. However, due to the large differences in the use and management of medical devices among hospitals, conventional disinfection management policies cannot be applied to every hospital and need to be adjusted according to the actual situation of the hospital to improve the quality of disinfection management. FMEA is a valuable reliability management tool that can identify potential failures of a system in advance and evaluate their causes and effects, thereby reducing the risk of potential failures and improving incident prognosis. Currently, FMEA is increasingly used in healthcare settings. It is widely popular and plays an important role in the fields of medical management, medical informatization, medical equipment and production (15, 16). Pirouz et al. (17) conducted a study using FMEA to identify and map 10 critical processes and 7 sub-processes in the operating room. They identified a total of 187 failure modes, which were scored based on their severity and probability. Based on these scores, they developed specific guidelines and monthly review procedures to address and mitigate risks, thereby reducing the likelihood of adverse events in the operating room environment. Establishing an individualized cleaning and disinfection management system based on FMEA, and adjusting and improving it in a timely manner according to individual conditions will help improve the quality of cleaning and disinfection of medical devices (18, 19). And with the rapid development of information technology in recent years, information management has gradually been applied in clinical practice. Information management not only plays an active role in organizing and maintaining electronic medical data, but also enables the sharing of medical data. The establishment of a cleaning and disinfection information management system based on registration information technology will help supervise the disinfection operation of medical devices and improve the efficiency of medical device management (20). In this work, the theoretical knowledge scores and operational skill scores of the staff in the research group were significantly higher than those in the control group. The pass rates of theoretical knowledge and operational skills of the control group and the research group were 44.64% 94.64and 96.43%, respectively. The pass rates of 55.36% theoretical knowledge and operational skills of the staff in the research group were significantly higher than those of the control group. The passing rates for theoretical knowledge and operational skills in the control group were 44.64 and 55.36%, respectively, while in the research group, they were 94.64 and 96.43%. The research group’s passing rates for both theoretical knowledge and operational skills were significantly higher compared to those of the control group. The RPN scores of medical staff factors, environmental factors, system factors, and institutional guarantee factors in the study group were significantly lower than those in the control group. The results show that management based on standardization and cleaning and disinfection information systems can improve the theoretical knowledge and operational skills of cleaning and disinfection personnel and reduce the risk of unqualified cleaning and disinfection. The results are similar to those of Yi L et al. (21). This study demonstrates that FMEA can be used to control the risk of surgical instrument packaging defects, effectively reduce the packaging defect rate and ensure patient safety.

Cleaning the surface of hospital instruments and equipment is an important part of disinfection and isolation, and it is also an important part of preventing hospital infections (22, 23). Standardizing the cleaning and disinfection operations of medical equipment will help effectively kill pathogens on the surface of the equipment, keep the equipment clean, and reduce the number of hospital infections. It can reduce infection while reducing instrument corrosion, helping to extend the service life of the instrument (24, 25). In this experiment, the qualified rates for cleaning and disinfection of instruments and equipment in the control group and the research group were 55.36 and 87.50%, respectively. The pass rate of cleaning and disinfection of instruments and equipment in the research group was significantly higher than that in the control group. It shows that management based on standardization and cleaning and disinfection information systems can effectively improve the quality of cleaning and disinfection of hospital instruments and equipment and reduce the incidence of unqualified cleaning and disinfection. The reason is that information management can effectively supervise the quality and frequency of cleaning and disinfection of medical devices, and at the same time, regular information-based cleaning and disinfection training can be conducted to improve employees’ operational capabilities (26, 27). FMEA cleaning and disinfection management can standardize the equipment placement area, formulate cleaning and disinfection standards for hospital equipment and equipment, and carry out targeted intervention and response to the risk factors of unqualified cleaning and disinfection. Standardizing cleaning and disinfection operations through systems and processes can help improve the quality of cleaning and disinfection (28, 29). In addition, this experiment found that the clinical satisfaction of the control group and the research group were 76.79 and 91.07%, respectively. The clinical satisfaction of the research group was significantly higher than that of the control group. This further proves that management based on standardization and cleaning and disinfection information systems can improve clinical satisfaction, the application effect is better in the aspect of “cleaning and disinfection of instruments and equipment” in hospitals.

In short, through standardization and management of cleaning and disinfection information systems, the theoretical knowledge and technical operation ability of cleaning and disinfection personnel can be effectively improved, the RPN score of disinfection and cleaning can be reduced, and the cleaning and disinfection effect and clinical satisfaction of instrument and equipment surfaces can be improved for promotion and application. However, due to the short study period of this experiment, the experimental results May be due to chance. In the future, we will expand the experimental subjects, increase the research time, and conduct in-depth exploration again.

## Data Availability

The raw data supporting the conclusions of this article will be made available by the authors, without undue reservation.
